# CT-based radiomics nomogram for differentiation of adrenal hyperplasia from lipid-poor adenoma: an exploratory study

**DOI:** 10.1186/s12880-022-00951-x

**Published:** 2023-01-07

**Authors:** Hongtao Yuan, Bing Kang, Kui Sun, Songnan Qin, Congshan Ji, Ximing Wang

**Affiliations:** 1 Shandong Provincial Hospital, Shandong University, Jinan, Shandong China; 2 Department of Radiology, Shandong Provincial Hospital Affiliated to Shandong First Medical University, Jinan, Shandong China; 3 School of Medicine, Shandong First Medical University, Jinan, Shandong China

**Keywords:** Adrenal hyperplasia, Adrenocortical adenoma, Tomography, X-ray computed, Nomograms

## Abstract

**Background:**

To establish and verify a radiomics nomogram for differentiating isolated micronodular adrenal hyperplasia (iMAD) from lipid-poor adenoma (LPA) based on computed tomography (CT)-extracted radiomic features.

**Methods:**

A total of 148 patients with iMAD or LPA were divided into three cohorts: a training cohort (n = 72; 37 iMAD and 35 LPA), a validation cohort (n = 36; 22 iMAD and 14 LPA), and an external validation cohort (n = 40; 20 iMAD and 20 LPA). Radiomics features were extracted from contrast-enhanced and non-contrast CT images. The least absolute shrinkage and selection operator (LASSO) method was applied to develop a triphasic radiomics model and unenhanced radiomics model using reproducible radiomics features. A clinical model was constructed using certain laboratory variables and CT findings. Radiomics nomogram was established by selected radiomics signature and clinical factors. Nomogram performance was assessed by calibration curve, the areas under receiver operating characteristic curves (AUC), and decision curve analysis (DCA).

**Results:**

Eleven and eight extracted features were finally selected to construct an unenhanced radiomics model and a triphasic radiomics model, respectively. There was no significant difference in AUC between the two models in the external validation cohort (0.838 vs. 0.843, *p* = 0.949). The radiomics nomogram inclusive of the unenhanced model, maximum diameter, and aldosterone showed the AUC of 0.951, 0.938, and 0.893 for the training, validation, and external validation cohorts, respectively. The nomogram showed good calibration, and the DCA demonstrated the superiority of the nomogram compared with the clinical factors model alone in terms of clinical usefulness.

**Conclusions:**

A radiomics nomogram based on unenhanced CT images and clinical variables showed favorable performance for distinguishing iMAD from LPA. In addition, an efficient unenhanced model can help avoid extra contrast-enhanced scanning and radiation risk.

## Background

An adrenal adenoma is the most common type of adrenal gland tumor. It is a benign tumor derived from the adrenal cortical tissue [1], usually presenting as a small rounded or oval mass (often measuring < 3 cm) [2]. Due to the existence of intrinsic fat content [3], more than 70% adrenal adenomas can be evidentially identified using a threshold of ≤ 10 Hounsfield units (Hu) on unenhanced computed tomography (CT), while those with > 10 Hu are classified into lipid-poor adenoma (LPA) [4–6].

Adrenal hyperplasia is a genetic disorder and physiologic overgrowth of adrenocortical tissue that affects the adrenal glands. It can be nodular or diffuse. Diffuse type manifests as a diffuse and smooth increase in the size of adrenal glands [6]; the nodular forms can be further classified into the primary pigmented micronodular adrenal type and the isolated micronodular hyperplasia (iMAD) type [7]. iMAD is characterized by a hyperplasia of the internodular tissue [8]. Patients with iMAD can be easily misdiagnosed with other high-attenuation diseases, such as LPA [3, 9]. The diagnosis of this condition is mainly based on conventional clinic and radiological methods, which are not sensitive enough to differentiate iMAD from LPA.

Patients with adrenal adenoma and adrenal hyperplasia can have similar clinical symptoms, including primary hypertension, primary aldosteronism, or Cushing's syndrome [10, 11]. Thus, differentiating these conditions may be challenging. While adrenal hyperplasia is resolved using a medication, patients with adrenal adenoma usually require surgery, i.e., adrenalectomy [12]. Adrenalectomy lowers blood pressure levels [13], ameliorates hypokalemia, reduces plasma aldosterone levels [14], and drastically increases survival in patients without atrial fibrillation [15]. On the other hand, the use of adrenalectomy in the management of adrenal hyperplasia is controversial. Surgery has been associated with certain side effects in these patients, including abnormal circadian rhythm [16] and corticotrophin deficiency [17]; also, these patients have a higher risk of having anesthesia accidents and extra medical expenses. Thus, accurate preoperative diagnosis is of utmost importance for therapy decision-making [18]. However, diagnosing based on conventional clinic and radiological methods remains challenging. Hence, a high-performance diagnostic approach needs to be established and certified.

Radiomics is a relatively new quantitative approach for medical imaging that extracts many features (e.g., intensity, geometry, and texture) from images using data-characterization algorithms. Over the years, this approach has been mainly applied in oncology but also in some other medical areas [19, 20]. Successful studies on adrenal tumors have been reported using radiomics to distinguish between benign adrenal tumors with malignant tumors [21] and pheochromocytoma with the other benign tumors [22, 23]. Yet, to our knowledge, no study have reported CT-based radiomics to distinguish iMAD from LPA. Thus, in this study, we developed and validated a radiomics nomogram incorporating the radiomics signature and the clinical factors for preoperative differential diagnosis of iMAD and LPA.

## Methods

### Patients

**Fig. 1:**
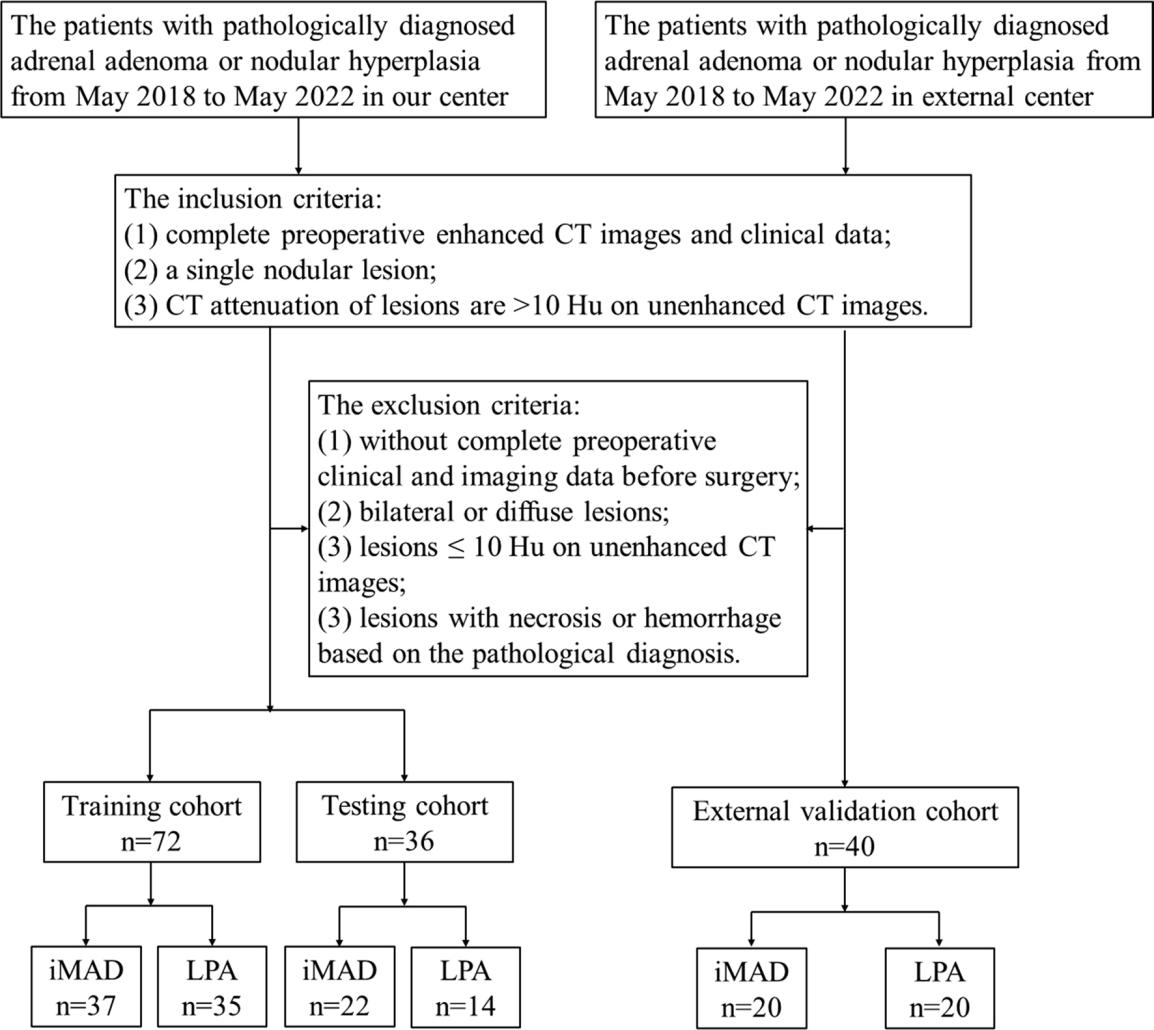
Study flow chart

Data for all cases were acquired from two medical centers: center 1 and center 2, from May 2018 to May 2022. Five hundred and sixty-eight patients who accepted unilateral adrenalectomy were included in the study, and they were all diagnosed with adrenal hyperplasia or adenoma pathologically. All patients underwent contrast-enhanced CT before surgery. CT images of all patients were further analyzed to identify iMAD or LPA using the following inclusion criteria: (1) patients with a single nodular lesion; (2) CT attenuation of lesions are > 10 Hu on unenhanced CT images. The exclusion criteria were: (1) patients with lesions ≤ 10 Hu on unenhanced CT images; (2) bilateral or diffuse lesions; (3) lesions with necrosis or hemorrhage based on the pathological diagnosis; (4) patients without complete preoperative clinical and imaging data before surgery. The patient recruitment pathway is presented in Fig. 1.

Finally, 148 patients were recruited: 108 patients from center 1 were divided into two cohorts (training and testing cohort) according to the ratio of 2:1 using computer-generated random numbers, and 40 patients from center 2 were incorporated into the external validation cohort.

The institutional review board of our hospital approved this retrospective study. Therefore, the requirement for obtaining informed consent was waived.

### CT image acquisition

In center 1, patients underwent CT scanning using multidetector-row CT systems (Ingenuity CT, Philips; Aquilion ONE, TOSHIBA; Somatom Force, Siemens Healthcare; Lispeed 64, GE Healthcare). The scanning parameters were as follows: 120 kV tube voltage; 250–400 mA tube current (automatic tube current modulation was applied); 0.5 s or 0.6 s rotation time; 192 × 0.6 mm or 64 × 0.625 mm detector collimation; a matrix of 512 × 512, and a pitch of 0.6 or 1.0. Axial images of 1 mm slice thickness were reconstructed. An 80–90 mL volume of iodinated contrast medium (Omnipaque 350, GE Healthcare, Shanghai, China) was injected via antecubital vein by a power injector (at a rate of 3.0 mL/s). Pre-enhanced CT images were acquired before two postcontrast CT scannning in the arterial phase (25–30 s) and the venous phase (60–70 s).

In center 2, patients were examined using the multidetector-row CT systems (Somatom Force, Siemens Healthcare; Discovery 750, GE Healthcare). The scanning parameters were as follows: 100 or 120 kV tube voltage; 0.5 or 0.6 s rotation time; 250–400 mA tube current (automatic tube current modulation was applied); 192 × 0.6 mm or 64 × 0.625 mm detector collimation; a matrix of 512 × 512, and a pitch of 1. Axial images of 1 mm slice thickness were reconstructed. An 80–90 mL volume of iodinated contrast medium (Iopromide, Ultravist 300; Bayer, Germany) was injected via antecubital vein using a power injector (at a rate of 2.5 mL/s). Unenhanced CT images were acquired before two postcontrast CT scanning in the arterial phase (25–30 s) and venous phase (55–60 s).

### Conventional CT feature evaluation

All of the image analysis were performed by the same radiology resident (Reader 1, HY) and a radiologist (Reader 2, CJ) with 5 and 10 years of abdominal imaging experience, respectively. The radiologists were blinded to the radiological reports and pathologic details. Reader 1 construed the following CT features by consensus: the maximum diameter (MD) and the CT attenuation of unenhanced phase (CT_pre_) and venous phase (CT_V_) on the axial CT image. All quantitative values were measured 3 times, and an average figure was applied.

### Clinical factors selection and construction of the clinical factor model

Clinical data included specific assay indexes (cortisol, aldosterone, and renin) and data regarding age, sex, and history of hypertension. All information were obtained from medical records. Univariate logistic analysis was used to compare the differences in the clinical factors (including clinical data and CT features) in the training cohort between the two groups; a multiple logistic regression analysis was applied to build the clinical model by using the significant variables from the univariate analysis as inputs. Odds ratios (OR) with 95% confidence intervals (CI) for each independent factor were calculated as relative risk estimates.

### Three-dimensional segmentation and radiomics feature extraction

**Fig. 2:**
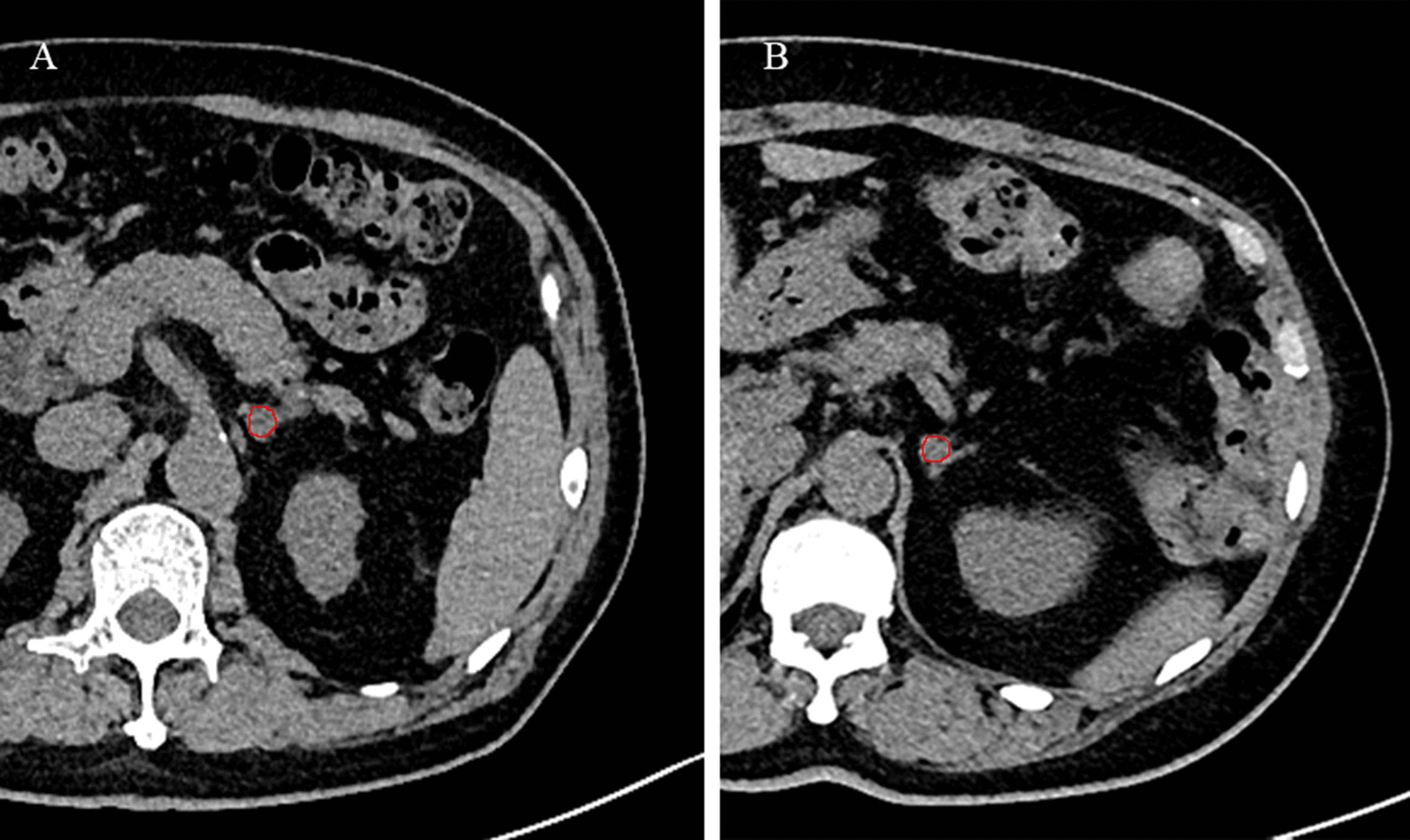
Manual segmentation of the lesion on the axial slice. **A** iMAD and **B** LPA

Image resampling was performed before feature extraction to decrease the variability of radiomics features. Images were resampled to 1 × 1 × 1 mm^3^ voxels using the B-Spline interpolation method. A soft tissue CT window was modified with a level of 40HU and a width of 300 Hu. Three-dimensional lesion segmentation was manually delineated as a region of interest (ROI) on images of three phases at ITK-SNAP (http://www.itksnap.org/pmwiki/pmwiki.php). Images of corresponding arterial and venous phases were used as reference when the contour on the unenhanced image was delineated. An example of manual segmentation is shown in Fig. 2.

Radiomics extraction was performed with Deepwise Research Portal (Deepwise Bolian, Co., Ltd.), integrated with PyRadiomics. A total of 1288 radiomics features were extracted from each phase. Twenty patients (10 iMAD and 10 LPA) were randomly chosen for further evaluation of feature stability: both the two radiologists repeated the same delineation procedure two weeks later. Inter- and intra- class correlation coefficients (ICC) were used to assess the extracted features’ inter-observer reliability and intra-observer reproducibility. It is commonly accepted that intra- and inter-class correlation coefficient (ICC) < 0.5 indicates poor reliability, 0.5–0.75: moderate reliability, and > 0.75: good or excellent reliability [24]. Thus, the features with ICC < 0.75 were excluded.

### Construction and comparison of the radiomics signatures

Z-score normalization was first used to standardize the features before the following analysis. Dimension reduction was conducted before signature construction in order to eliminate features overfitting. The stable radiomics features were assessed by one-way analysis of variance, and the features that were significantly different between the two groups (*p* < 0.05) were enrolled into the least absolute shrinkage and selection operator (LASSO) regression model in order to select the most valuable features in the training cohort. Then, the selected features were applied to build radiomics signatures. Two radiomics models were established: a triphasic CT feature based on unenhanced and enhanced CT features and an unenhanced radiomics model based on unenhanced CT features. A radiomics score (Rad-score) was calculated for each patient through a linear combination of selected features weighted by their respective LASSO coefficients.

### Development of a radiomics nomogram and assessment of the performance of different models

Delong’s test was first used to compare two radiomics models before the radiomics nomogram was performed. The triphasic radiomics model was chosen when establishing a nomogram if there was a significant difference between two radiomics signatures; otherwise, an unenhanced radiomics model was chosen. Then, a radiomics nomogram was establish by incorporating the chosen radiomics model and the selected clinical factors. A radiomics nomogram score (Nomo-score) was calculated for each patient. The calibration of the nomogram was verified using a calibration curve. The Hosmer–Lemeshow test was used to validate the goodness-of-fit of the nomogram. The diagnostic performance of the clinical model, the chosen radiomics signature, and the radiomics nomogram was assessed based on the area under the receiver operator characteristic curve (AUC) in the training, testing, and external validation sets. Decision curve analysis (DCA) was performed to evaluate the clinical usefulness of three models by calculating the net benefits for a range of threshold probabilities in the external validation cohort [21].

### Statistical analysis

Statistical tests were performed using MedCalc statistical software (version 20.110, https://www.medcalc.org), R statistical software (version 3.3.3, https://www.r-project.org), and MATLAB (version 2021a). Student’s t-tests or non-parametric tests (where appropriate) were used for continuous variables. Chi-squared test or Fisher’s exact test (where appropriate) were used for categorical variables. Categorical and continuous variables are shown in frequency (percentages), mean ± standard deviation, or median (interquartile range), where appropriate. The AUC was compared by Delong’s test. A *p* value < 0.05 was considered to be statistically significant.

## Results

### Clinical baseline and construction of the clinical model

The patients’ demographic baseline characteristics are shown in Table 1. The rates of LPA in the training, testing, and external validation cohort were 48.6% (35 of 72), 38.9% (14 of 36), and 50.0% (20 of 40), respectively. The results of univariate and multiple logistic regression analysis are listed in Table 2. Multiple logistic regression showed that maximum diameter and aldosterone levels are independent predictors of LPA (all *p* < 0.05), thus were chosen to create a clinical model. Lesions with larger maximum diameter (OR, 1.296; 95% CI, 1.036–1.621) or higher aldosterone (OR, 1.005; 95% CI, 1.000 -1.010) were likely to be LPA.

**Table 1:** Clinical and conventional CT radiological features of all cohorts

Variable	Training cohort			Testing cohort			External validation cohort		
	iMAD n = 37	LPA n = 35	*p* value	iMAD n = 22	LPA n = 14	*p* value	iMAD n = 20	LPA n = 20	*p* value
Age (years)	53.81 ± 1.93	53.4 ± 1.59	0.871^a^	56.14 ± 2.12	50.21 ± 2.33	0.077^a^	53.70 ± 2.70	44.73 ± 2.42	0.071^a^
Sex			0.984^b^			1.000^b^			0.749^b^
Male	20 (54.10%)	19 (54.30%)		11 (50.00%)	7 (50.00%)		11 (55.0%)	12 (60.0%)	
Female	17 (45.90%)	16 (45.70%)		11 (50.00%)	7 (50.00%)		9 (45.0%)	8 (40.0%)	
Hypertension			0.023^b^			0.956^b^			0.465^b^
Yes	26 (70.30%)	32 (91.40%)		19 (86.40%)	12 (85.70%)		15 (80.0%)	14 (70.0%)	
No	11 (29.70%)	3 (8.60%)		3 (13.60%)	2 (14.30%)		5 (20.0%)	6 (30.0%))	
Cortisol (nmol/L)	330.05 ± 17.70	306.66 ± 17.50	0.427^a^	331.33 ± 29.09	318.10 ± 30.53	0.587^a^	347.31 ± 27.22	308.98 ± 30.61	0.355^a^
Renin (pg/mL)	8.12 [5.49, 16.52]	6.95 [2.58, 15.56]	0.382^c^	11.57 [6.52, 22.79]	3.29 [1.27, 22.79]	0.020^c^	8.98 [6.24, 16.39]	9.95 [6.85, 15.3]	0.829^c^
Aldosterone (pg/mL)	158.86 [122.63, 210.15]	191.68 [123.21, 358.67]	0.068^c^	165.99 [117.6, 194.57]	234.10 [111.38, 407.11]	0.269^c^	154.98 [104.31, 188.13]	209.54 [141.50, 307.37]	0.024^c^
Sodium (mmol/L)	140.71 ± 2.19	141.32 ± 1.98	0.227^a^	140.02 ± 1.72	141.02 ± 1.24	0.103^a^	140.54 ± 1.58	141.46 ± 1.72	0.083^a^
Potassium (mmol/L)	4.04 ± 0.37	4.16 ± 0.41	0.210^a^	3.81 ± 0.43	4.05 ± 0.39	0.083^a^	4.02 ± 0.49	3.99 ± 0.52	0.877^a^
Albumin (g/mL)	42.90 [40.90, 44.60]	41.60 [39.70, 45.50]	0.358^c^	42.05 [40.38, 44.58]	41.65 [39.50, 44.33]	0.685^c^	43.41 ± 3.19	44.53 ± 2.65	0.174^c^
MD (mm)	8.90 ± 0.50	10.45 ± 0.40	0.019^a^	7.41 ± 0.37	9.76 ± 0.95	0.012^a^	9.12 ± 0.78	9.57 ± 0.51	0.641^a^
CT_pre_(Hu)	25.53 ± 1.50	26.40 ± 1.80	0.711^a^	27.60 ± 1.78	27.64 ± 1.59	0.989^a^	29.41 ± 1.87	24.65 ± 2.98	0.093^a^
CT_V_(Hu)	87.98 ± 3.25	80.31 ± 4.19	0.035^a^	92.11 ± 6.77	85.06 ± 4.91	0.115^a^	90.29 ± 4.94	77.9 ± 6.38	0.087^a^

*MD* maximum diameter, *CT*_*pre*_ CT attenuation of unenhanced phase, *CT*_*V*_ CT attenuation of venous phase

^a^Statistical analysis performed using independent-samples T test

^b^Statistical analysis performed using chi-square test

^c^Statistical analysis performed using Mann–Whitney U test

**Table 2:** Univariate and multiple logistic regression analyses of clinical factors

Variable	Univariate analysis		Multiple analysis	
	OR (95% CI)	*p* value	OR (95% CI)	*p* value
Age	0.869 (0.954–1.041)	0.869		
Sex	1.009 (0.399–2.552)	0.984		
Hypertension	4.513 (1.138–17.893)	0.032*	3.932 (0.833–18.569)	0.084
Cortisol	0.998 (0.993–1.002)	0.348		
Renin	1.004 (0.965–1.044)	0.850		
Aldosterone	1.005 (1.001–1.010)	0.020*	1.005 (1.000–1.010)	0.036**
Sodium (mmol/L)	1.154 (0.915–1.455)	0.227		
Potassium (mmol/L)	2.203 (0.642–7.563)	0.210		
Albumin (g/mL)	0.974 (0.907–1.045)	0.463		
MD	1.249 (1.028–1.517)	0.025*	1.296 (1.036–1.621)	0.023**
CT_pre_	1.009 (0.962–1.058)	0.707		
CT_V_	0.984 (0.962–1.006)	0.155*	0.094 (0.970–1.019)	0.647

*MD* Maximum diameter, *CT*_*pre*_ CT attenuation of unenhanced phase, *CT*_*V*_ CT attenuation of venous phase, *OR* Odd ratio, *95%CI* 95% Confidence interval

*p < 0.2 was selected for multiple logistic regression analysis

**p < 0.05 was selected as independent predictive factors

### Feature extraction, selection, and radiomics signature establishment and comparison

**Fig. 3:**
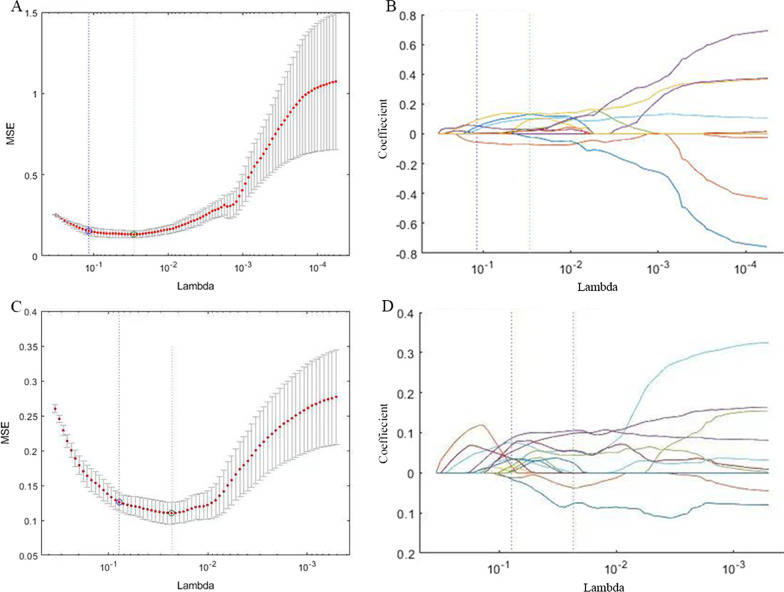
Feature selection and cross-validation using LASSO. For different values of lambda, different numbers of features will be included in the final model and a decision has to be made. Figures A, C illustrate the process of cross validation in order to choose the most appropriate lambda value. Lambda resulting in the smallest mean squared error (MSE) is chosen, as the green lines shows. Figures **B**, **D** plot different values of lambda on the x-axis and each line represents a feature, showing when it enters the model and its level of influence on the outcome. **A** Selection of the tuning parameter (lambda) in the LASSO of unenhanced model. **B** The unenhanced CT feature coefficients varied according to lambda. **C** Selection of the tuning parameter (lambda) in the LASSO of enhanced model. **D** The enhanced CT feature coefficients varied according to lambda

A total of 710 unenhanced CT radiomics features, 863 arterial CT features and 707 venous CT features were considered robust features (ICCs > 0.75). Sixteen triphasic features and eleven unenhanced features were selected by LASSO logistic regression model to construct the triphasic radiomics model and the unenhanced radiomics model, respectively (Fig. 3).

For the unenhanced model, radiomics score [median (interquartile range)] differed significantly between iMAD and LAP groups in the training cohort [− 0.11 (− 0.15, − 0.03) vs. 0.10 (0.04, 0.15), respectively, *p* < 0.001]; this was further verified in the validation cohort [− 0.07 (− 0.12, 0,03) vs. 0.07 (0.01, 0.19), respectively, *p* < 0.001] and the external validation cohort [− 1.74 (− 4.14, − 0.38) vs. 0.65 (0.22, 4.54) respectively, *p* < 0.001]. The unenhanced model yielded an AUC of 0.916 (95% CI 0.826, 0.968) in the training cohort, 0.860 (95% CI 0.704, 0.953) in the validation cohort and 0.838 (95% CI 0.687, 0.935) in the external validation cohort, showing favorable discriminative efficacy. The triphasic radiomics model was also identified as a good classifier, yielding an AUC of 0.964, 0.880, and 0.843 in three cohorts, respectively.

**Fig. 4:**
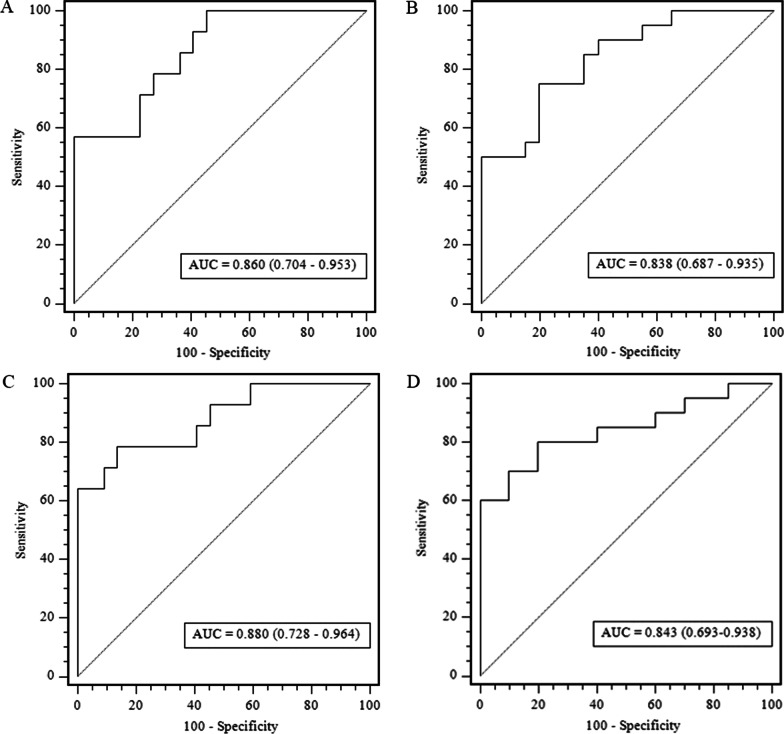
Performance of the unenhanced model and triphasic model. Receiver operating characteristic (ROC) curves of the radiomics signature in the testing and external validation cohorts of the unenhanced model **A**, **B** and triphasic model **C**, **D**, respectively. AUC, area under the receiver operating characteristic curve

Delong’s test was performed for comparison. No significant differences were found between the unenhanced and triphasic models (*p* = 0.949). The ROC analysis of two radiomics models in testing and external validation cohort is shown in Fig. 4. The AUC, 95% CI of AUC, sensitivity, specificity, accuracy, and a cut-off value are shown in Table 3. The radiomics score of unenhanced radiomics model was calculated by following formula: “Rad-score = 0.1324 × original_shape_LeastAxisLength + 0.1302 × gradient_glcm_ClusterTendency + 0.1020 × wavelet-HLH_firstorder_Kurtosis + 0.0950 × wavelet-LHH_glrlm_LongRunLowGrayLevelEmphasis + 0. 0322 × square_glcm_Idmn + 0.0247 × wavelet-LLH_glrlm_RunEntropy + 0.0201 × wavelet-LHH_firstorder_Range + 0.0189 × wavelet-HLH_firstorder_Range − 0.0219 × wavelet-LHH_firstorder_Minimum − 0.0323 × squareroot_gldm_SmallDependenceEmphasis − 0.0707 × original_firstorder_Skewness + 0.4861”.

**Table 3:** Results of unenhanced radiomics model and triphasic radiomics model predictive ability for distinguishing between iMAD and LPA

Model		AUC (95% CI)	Sensitivity (%)	Specificity (%)	Accuracy (%)	Cut-off
Unenhanced radiomics model	Training cohort	0.916 (0.826–0.968)	88.6	91.9	90.3	> 0.513
	Testing cohort	0.860 (0.704–0.953)	78.6	72.7	72.2	
	External validation cohort	0.838 (0.687–0.935)	75.0	80.0	77.5	
Triphasic radiomics model	Training cohort	0.964 (0.892–0.994)	91.4	91.9	93.1	> 0.483
	Testing cohort	0.880 (0.728–0.964)	78.6	86.3	80.6	
	External validation cohort	0.843 (0.693–0.938)	80.0	80.0	77.5	

*AUC* Area under the receiver operator characteristic curve, *95% CI* 95% Confidence interval

### The radiomics nomogram establishment and assessment of the performance of different models

**Fig. 5:**
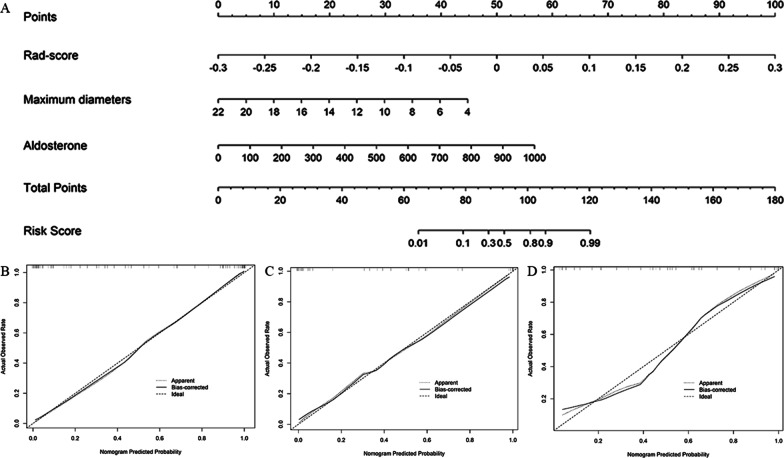
Radiomics nomogram and calibration curves. **A** The radiomics nomogram, combining maximum diameter, aldosterone and Rad-score, was developed in the training set. **B**–**D** The nomogram calibration curves in training (**B**), testing (**C**), and external validation (**D**) sets. Calibration curves indicate the goodness-of-fit of the model

Aldosterone count, maximum diameter, and Rad-score (unenhanced radiomics model) were incorporated to develop a radiomics nomogram in the training cohort (Fig. 5a). The calibration curve of the radiomics nomogram demonstrated good agreement between the predicted and expected probabilities in three cohorts (Fig. 5b–d). The *p* values of Hosmer–Lemeshow test in training, validation, and external validation cohorts were 0.077, 0.424, and 0.410, respectively.

**Fig. 6:**
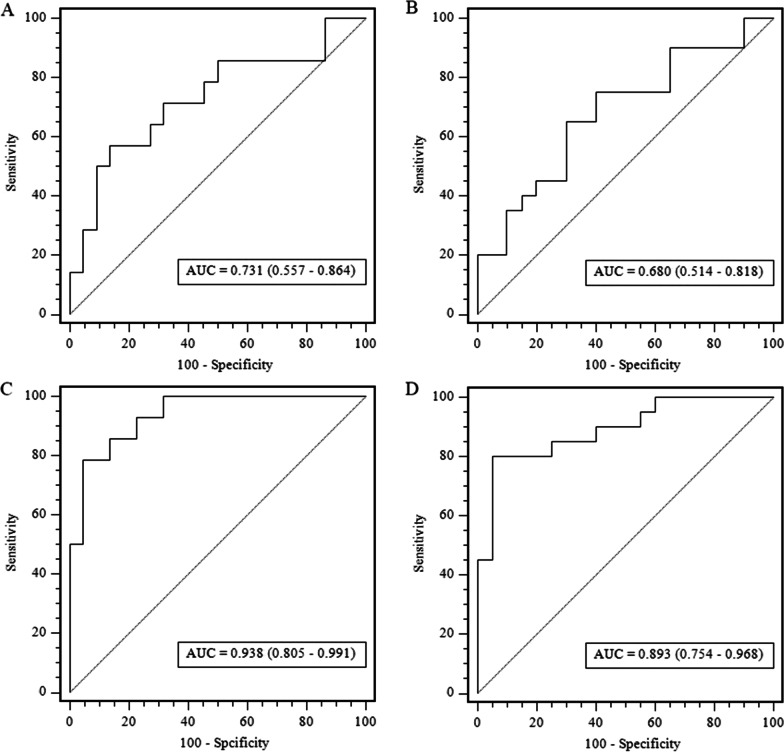
Performance of the clinical model and the radiomics nomogram. Comparison of receiver operating characteristic (ROC) curves of the clinical model (**A**, **B**) and radiomics nomogram (**C**, **D**) for the prediction of iMAD and LPA in the testing and external validation cohorts, respectively. AUC, area under the receiver operating characteristic curve

The diagnostic performance of each model is shown in Table 4. The ROC curves of the clinical model and radiomics nomogram are presented in Fig. 6. The radiomics nomogram showed the highest discrimination in the training cohort, with an AUC of 0.951 (95% CI 0.873, 0.998), and also achieved satisfactory predictive efficacy in the validation cohort [AUC, 0.938 (95% CI 0.805, 0.980)] and in the external validation cohort [AUC, 0.893 (95% CI 0.754, 0.968)]. The observed AUC value was higher than that of the clinical model in external validation [AUC, 0.680 (95% CI 0.514, 0.818); *p* = 0.030]. The nomo-score was acquired using the following formula: “Nomo- score = 27.5663 × Rad-score − 0.4115 × maximum diameter + 0.0094 × aldosterone + 2.0275”.

**Table 4:** Results of unenhanced model and triphasic model predictive ability for distinguishing between iMAD and LPA

Model		AUC (95% CI)	Sensitivity (%)	Specificity (%)	Accuracy (%)	Cut-off
Clinical model	Training cohort	0.764 (0.650–0.857)	74.3	70.3	70.8	> − 0.226
	Testing cohort	0.731 (0.557–0.864)	57.1	86.4	72.2	
	External validation cohort	0.680 (0.514–0.818)	75.0	60.0	67.5	
Unenhanced radiomics model	Training cohort	0.916 (0.826–0.968)	88.6	91.9	90.3	> 0.513
	Testing cohort	0.860 (0.704–0.953)	78.6	72.7	72.2	
	External validation cohort	0.838 (0.687–0.935)	75.0	80.0	77.5	
Radiomics nomogram	Training cohort	0.951 (0.873–0.988)	85.7	97.3	91.7	> 0.363
	Testing cohort	0.938 (0.805–0.980)	78.6	95.5	88.5	
	External validation cohort	0.893 (0.754–0.968)	80.0	95.0	87.5	

*AUC* Area under the receiver operator characteristic curve, *95% CI* 95% Confidence interval

**Fig. 7:**
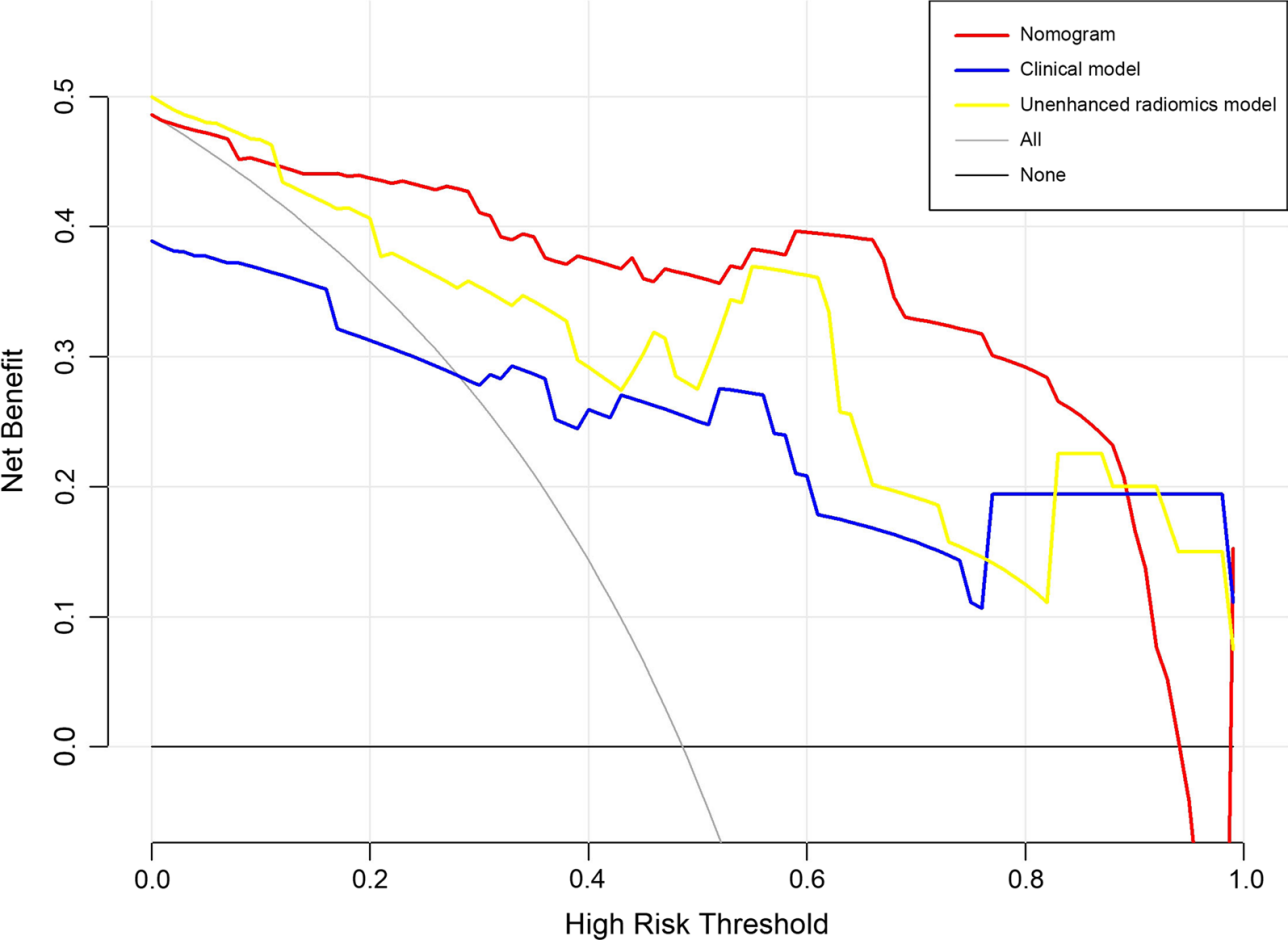
Decision curve analysis for different models. The y-axis shows the net benefit; the x-axis shows the threshold probability. The decision curves indicate that applying a radiomics nomogram to predict LPA adds great benefit than treating all or none of the patients across the large range of reasonable threshold probabilities

The decision curve analyses for the clinical model, unenhanced radiomics model, and radiomics nomogram in the external validation are presented in Fig. 7. Radiomics nomogram had the highest overall net benefit in a large range of threshold probability.

## Discussion

Our study established a radiomics nomogram that includes two clinical factors and an unenhanced radiomics signature for preoperative discrimination of iMAD and LPA. The proposed nomogram had better diagnostic performance than the clinical model alone or radiomics signature in differentiating iMAD from LPA. This nomogram can further the treatment decision-making and improve clinical outcomes.

In clinical practice, an isolated adrenal nodule can be incidentally observed in a routine abdominal or chest CT scan. A nodule that has a smaller (generally ≤ 4 cm) size, higher attenuation (generally ≥ 20Hu), or no progression during a follow-up visit can be used as an indicator of the benign lesion [25]. However, the differential diagnosis between small benign adrenal lesions can be challenging, considering that patients may present similar symptoms, including hypokalemia, hypertension, and aldosteronism. Due to the lack of fat contents, LPA presents atypical features and can be easily misdiagnosed with iMAD [3, 9]. However, accurate diagnosis is extremely important considering that patients with LPA and iMAD require different treatment approaches [12]. Surgery in patients with adrenal hyperplasia has been associated with certain side effects [16]. The benefit of surgery performed on iMAD patients is still indeterminate.

To our knowledge, only a few studies have investigated the difference between adrenal adenoma and hyperplasia. Leung et al. [26] created a clinical prediction score using 3 parameters (plasma renin activity before saline infusion in saline infusion test ≤ 0.26 ng/mL/h, age at diagnosis < 50 years, and aldosterone level after saline infusion in saline infusion test ≥ 424 pmol/L) to predict aldosterone‑producing adenoma from idiopathic adrenal hyperplasia. Other clinical prediction criterion also emphasized the effect of aldosterone [27]. This is consistent with our result that patients with LPA had higher aldosterone levels and aldosterone was an independent predictor. Park et al*.* analyzed several CT parameters, pointing out significant differences in lesion size between adrenal adenoma and adrenal hyperplasia [12]. In the present study, we found that maximum diameter was an independent predictive factor and LPA is larger than iMAD. CT attenuation of the venous phase can serve as a potential risk predictor. However, multiple logistic regression analysis suggested that CT attenuation of the venous phase is not an independent risk predictor. This is proved by other researches. Triphasic CT attenuation and percent washout could not be used for differentiation [12]. And an MRI-based study demonstrated that the quantitative evaluation of washout parameters is not sensitive enough to differentiate LPA from other non-adenomas [9]. However, Seo et al. [1] reported differences in absolute percent washout and relative percent washout between adenoma and non-adenoma, which contradicts aforementioned and our researches. This indicates that heterogeneity may exist based on traditional image factors.

Radiomics has been widely applied in the field of adrenal tumors. For example, texture analysis was used for differentiating malignant from benign adrenal tumors [21]. Furthermore, Chen et al. [28] and He et al. [29] used radiomics signature and nomogram to confirm the risk of functional adenoma. Moreover, radiomics can be an effective medical approach for predicting subclinical pheochromocytoma from lipid-poor adenoma [22]. In this study, radiomics was used to differentiate iMAD from LPA. Diffuse adrenal hyperplasia and adenoma with fat content can easily be identified by most radiologists, thus were excluded in this study. A nomogram was established and yielded a higher AUC than the clinical model did, showing a favorable clinical application value.

In this study, radiomics features concerning shape, grey level, and texture heterogeneity conduced to the radiomics signature. ‘original_shape_LeastAxisLength’ indicates that tumor size is an important factor. This is consistent with our discovery that size is of great importance. It was also used in another research explaining difference of lesions’ shape [30]. ‘wavelet-HLH_firstorder_Kurtosis’ is histogram-based and represented the position of peak height that indicates CT attenuation value of the maximum number of voxels [31]. A more homogeneous pixel distribution reflected a more regular nodular architecture. In addition, ‘original_firstorder_Skewness’ represented pixel asymmetry, indicating different heterogeneity of two kinds of nodules. This was also proved in Caruso et al*.*’s research as malignant tumor had higher heterogeneity [32]. Briefly, features of first-order and texture (GLCM, GLSZM, etc.) features reflected tumor heterogeneity and microenvironment, which is consistent with other studies [33–35].

We developed two radiomics signatures: an unenhanced radiomics model deriving from unenhanced CT features and a triphasic radiomics model deriving from unenhanced and enhanced CT features. Both models showed satisfying differential ability. Delong’s test indicated no significant difference between the two signatures, which implied that enhanced CT images might fail to provide more necessary texture information compared to unenhanced CT. Whether enhanced radiomics signatures are useful seems to be dubious and needs reconsidering. Yi and colleagues [22] observed the similar diagnostic efficacy of the unenhanced model and enhanced model in differentiating subclinical pheochromocytoma from adrenal lipid-poor adenoma. Sui et al. [36] found that the unenhanced CT model showed higher accuracy, sensitivity, and specificity in distinguishing high-risk anterior mediastinal lesions. He and his team [37] obtained identical conclusions when examining lung diseases. The above-mentioned studies seem to be supportive of our conclusion. The possible interpretation was that contrast enhancement was related to angiogenic properties of tumors [38] and both two diseases are rich in blood supply. So, the minor difference of angiogenic property under the microscope between iMAD and LPA was not powerful enough to provide more favorable details in regard to the difference in vascularity for the enhanced model. Moreover, the biological heterogeneity within the tumor depicted by radiomics features may be confounded by the intravenously injected contrast material, which may have trivial benefits in discrimination between iMAD and LPA. In brief, the cost-effectiveness of the enhanced-CT should be accurately assessed and the potential risks of an additional CT scan, such as radiation hazards and the potential risks associated with contrast media (allergy, potential renal damage and so on), should be considered. The risks related to contrast agent have been reported to be exacerbated in the pediatric and elderly populations.

This study has a few limitations. First, this was a retrospective study with small sample size; training and validation groups were also derived from one institute. Second, owing to the inherent deficiency of retrospective research, the CT images were from different scanners and parameters, which may influence the texture analysis. Also, resampling was arranged to minimize the effect as much as possible. Finally, 3-D manual ROI segmentation can be time-consuming and complicated to perform. Accordingly, an automatic segmentation method for adrenal lesions with favorable reliability and reproducibility needs to be developed and applied.

## Conclusions

In this study, we developed a CT-based radiomics nomogram that has favorable predictive effectiveness for preoperative differentiation of iMAD from LPA. Unenhanced CT protocol is of favorable clinical use to avoid external radiation and potential contrast agent risk. As a non-invasive and quantitative method, radiomics nomogram may serve as an effective tool to supplement the conventional imaging modalities for the clinical decision-making process.

## Data Availability

The datasets used and analysed during the current study available from the corresponding author on reasonable request.

## Abbreviations

iMAD: Isolated micronodular adrenal hyperplasia
LPA: Lipid-poor adenoma
LASSO: Least absolute shrinkage and selection operator
ROC: Receiver operator characteristic curve
DCA: Decision curve analysis
AUC: Areas under receiver operating characteristic curves
OR: Odds ratios
CI: Confidence intervals
ICC: Inter- and intra- class correlation coefficients
